# Supplementary material for: An Inverted Honeycomb Plasmonic Lattice as an Efficient Refractive Index Sensor
Source: Nanomaterials (Basel). 2021 May 4;11(5):1217. doi: 10.3390/nano11051217 (PMC8147928; doi:10.3390/nano11051217)
Supplement: Supplementary file 1 [file nanomaterials-11-01217-s001.zip › nanomaterials-1197902-supplementary.pdf]

Supplementary Materials

An inverted honeycomb plasmonic lattice as an efficient refractive index sensor

Javier Rodríguez-Álvarez[1,2,*], Lorenzo Gnoatto[1], Marc Martinez-Castells[1], Albert Guerrero[3], Xavier Borrisé[4], Arantxa Fraile Rodríguez[1,2], Xavier Batlle[1,2] and Amílcar Labarta[1,2]

Departament de Física de la Matèria Condensada, Universitat de Barcelona, 08028 Barcelona, Spain
[2] Institut de Nanociència i Nanotecnologia (IN2UB), Barcelona, 08028, Spain
[3] Institut de Microelectrónica de Barcelona (IMB-CNM, CSIC), Bellaterra, 08193, Spain
[4] Catalan Institute of Nanoscience and Nanotechnology (ICN2), CSIC and The Barcelona Institute of Science and Technology, Campus UAB, Bellaterra, 08193 Barcelona, Spain
* E-mail:javier.rodriguez@ub.edu

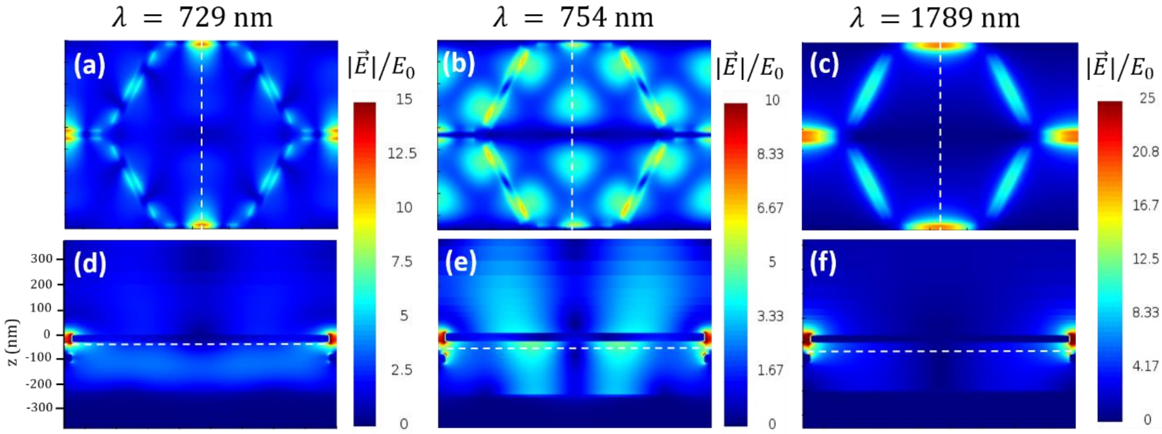

Figure S1. Near-field distributions for the peaks at 729 nm (a) and (d), 754 nm (b) and (e), and 1789 nm (c) and (f). (a), (b), and (c) correspond to in-plane views at the heights indicated by the dashed lines in the corresponding panels (d), (e), and (f). (d), (e), and (f) depict transversal cross sections of the heterostructure along the dashed lines plotted in the corresponding in-plane view panels (a), (b) and (c), respectively. They share a common vertical scale. $|\vec{E}|/E_0$ stands for the normalized modulus of the electric field with respect to the modulus of the incident radiation.

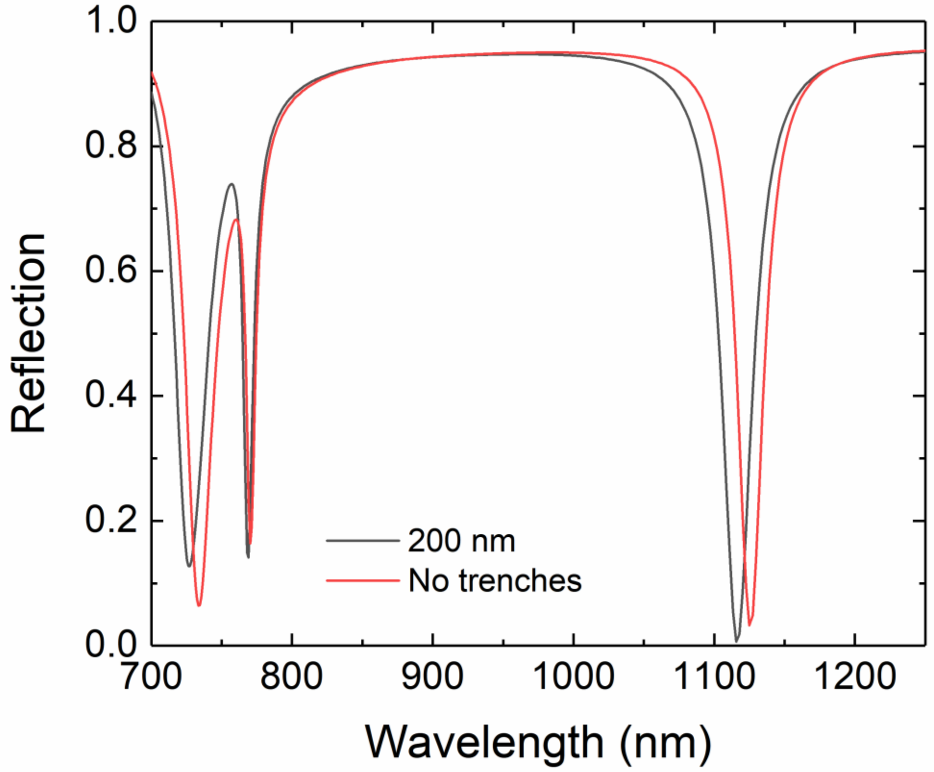

Figure S2. Reflection spectra for a heterostructure without the direct Au honeycomb lattice for the following two cases: a system with trenches carved through the whole thickness of 200 nm of the SiO_2 layer within the top-Au and mirror layers (black solid line) and a system with no trenches and a continuous SiO_2 layer (red solid line). The rest of the parameters are the same as those for the heterostructure in Figure 1.
